# Comparison of Graft Materials in Multilayer Reconstruction with Nasoseptal Flap for High-Flow CSF Leak during Endoscopic Skull Base Surgery

**DOI:** 10.3390/jcm11226711

**Published:** 2022-11-13

**Authors:** Byung Kil Kim, Doo-Sik Kong, Do-Hyun Nam, Sang Duk Hong

**Affiliations:** 1 Department of Otorhinolaryngology-Head and Neck Surgery, Kyungpook National University Chilgok Hospital, School of Medicine, Kyungpook National University, Daegu 41404, Korea; 2 Department of Neurosurgery, Samsung Medical Center, Sungkyunkwan University School of Medicine, Seoul 06351, Korea; 3 Department of Otorhinolaryngology-Head and Neck Surgery, Samsung Medical Center, Sungkyunkwan University School of Medicine, Seoul 06351, Korea

**Keywords:** acellular dermal matrix, cerebrospinal fluid leak, fascia lata, skull base, surgery

## Abstract

Cerebrospinal fluid (CSF) leak is a crucial complication after endoscopic skull base surgery. Therefore, multilayer reconstruction with grafts is as essential as a reconstruction with pedicled flaps. Although widely used, the multilayer technique with autologous fascia lata has drawbacks, such as additional wound and donor site complications. We compared acellular dermal graft and banked homologous fascia lata graft (alternative grafts) with autologous fascia lata graft for high-flow CSF leak repair. We retrospectively enrolled 193 subjects who underwent endoscopic skull base reconstruction with multilayer fascial grafts and nasoseptal flap for high-flow CSF leaks from November 2014 to February 2020 at a single institution. Acellular dermal matrix (ADM), banked homologous fascia lata, and autologous fascia lata were used in 48 (24.9%), 102 (52.8%), and 43 (22.3%) patients, respectively. Postoperative CSF leaks occurred in 23 (11.9%) patients and meningitis in 8 (4.1%). There was no significant difference in postoperative CSF leak (*p =* 0.36) and meningitis (*p =* 0.17) across the graft groups. Additionally, we could not find out contributing risk factors for postoperative CSF leak and meningitis. ADM and banked homologous fascia lata are non-inferior to autologous fascia lata for endoscopic skull base reconstruction in water-tight reconstruction or safety without additional donor site morbidities.

## 1. Introduction

Postoperative cerebrospinal fluid (CSF) leak is a possible complication of the transsphenoidal approach (TSA). In particular, when removing tumors involving the suprasellar area, posterior fossa, or anterior skull base through the endoscopic endonasal approach (EEA), high-flow CSF leak is inevitable [1]. The nasoseptal flap (NSF) has been used as a workhorse local flap for skull base reconstruction when high-flow CSF leak occurs during EEA [2,3], and multilayer reconstruction methods using various grafts have been introduced and considered to be important as an NSF [1,4,5]. In addition, synthetic materials, such as fibrinogen-based collagen fleece, fibrin glue, and gelatin foams, or autologous fat can play a supplementary role [6,7]. Usually, inlay and onlay graft techniques using autologous fascia lata, whose characteristics are similar to those of dura, have been performed, and the “gasket-seal” method using a rigid buttress with a fascial graft is also widely used for dura reconstruction [5,8,9]. However, after harvesting autologous fascia lata, additional scars or various wound problems, such as hematoma formation or herniation of muscle belly with pain, can occur. Instead, other graft materials, such as acellular dermal matrix (ADM) and allograft for fascia lata, have been used for dura reconstruction [10,11]. For example, homologous radiation-sterilized fascial grafts from cadaveric fascia lata can be used just as autologous fascia lata is used, and they have the advantage of avoiding scarring and other complications after harvesting [12]. However, there have been concerns that non-autologous grafts could not adhere to dura well or could be an infection source. Therefore, following endoscopic skull base reconstruction using a multilayer graft with a NSF, we analyzed the rate of postoperative CSF leak and meningitis in relation to the three different graft materials.

## 2. Materials and Methods

### 2.1. Study Subjects

We retrospectively investigated the medical data of 1395 consecutive patients who underwent EEA procedures with skull base reconstruction between November 2014 and February 2020 in a single institution. EEA was performed to remove the various types of tumors invading the sellar to suprasellar portion, posterior fossa, or anterior skull base. Among them, 193 patients who had an intraoperative high-flow CSF leak and underwent multilayer reconstruction using fascial grafts with NSF were included in this study. High-flow CSF leak was defined as the occurrence of large diaphragmatic/dural defect during resection through suprasellar, transclival or transcribriform approaches according to Esposito’s grade 3 [13]. Three types of grafts were used: acellular dermal matrix (ADM), banked homologous fascia lata, and autologous tensor fascia lata. Cases of using a graft or flap for spontaneous CSF leak repair rather than tumor resection were excluded. Additionally, patients diagnosed with Rathke’s cleft cyst at the final pathology were excluded to avoid a pivotal effect on the outcome. Postoperative meningitis was considered as follows: (1) patient had clinical symptoms of meningitis such as fever, headache, and/or meningeal irritation signs; (2) positive CSF cultures, and/or low glucose level along with increased white blood cell count and elevated levels of protein in the CSF. This retrospective study was approved by the Institutional Review Board (IRB) of Samsung Seoul Hospital (SMC 2022-06-008).

### 2.2. Graft Preparation

The three types of grafts were chosen depending on the time of surgery, the tissue bank situation or the patient’s preferences. We preferred the autologous fascia lata graft in the early study period (until 2015). After then, homologous fascia lata was chosen as the first option to avoid additional donor site morbidities. If there were not enough banked fascia lata in the tissue bank in our hospital, we used ADM and, if a patient had a preferred graft type, this was selected. The selection of graft material was chiefly determined after preoperative counselling, and informed consent was obtained from the patients prior to surgery regarding the availability of one of three types of grafts.

This study used cross-linked ADM as an allograft material, Megaderm^®^ (L&C Bio, Seoul, Korea) or Alloderm^®^ (LifeCell Corp, The Woodlands, TX, USA), made by processing donated human skin. Homologous fascia lata from the tissue bank in our hospital was used from harvesting cadaver donors’ tensor fascia lata. None of the donors had any transmissible viral disease based on screening tests. All tissues were sterilized by gamma irradiation (25 × 10^3^ Gy) and frozen at −70 °C in liquid nitrogen. An autologous graft was harvested at the patient’s lateral thigh for tensor fascia lata.

### 2.3. Reconstruction Techniques

All the reconstruction was performed by single ENT surgeon and two different neurosurgeons with two layered graft and nasoseptal flap. After the tumor was resected, the intradural space was gently covered with Surgicel^®^ (Ethicon, North Ryde, NSW, Australia) or fibrinogen collagen sponges (Tachosil^®^, Nycomed, Linz, Austria). If the intradural dead space was large, in case of olfactory groove meningioma or large posterior fossa defects, an abdominal fat graft was used to avoid dead space. Double-layered dura reconstruction using ADM, homologous fascia lata or autologous fascia lata was performed in an inlay–onlay fashion. The inlay graft was designed to be larger than the defect size in a rectangular shape and placed on the subdural surface. Then, the onlay graft was inserted into the epidural surface and under the skull base bone. An NSF was then applied to sufficiently cover the skull base defect and direct contact with the bare bone. Compressive packing was performed with synthetic materials, such as gelatin foams and Merocel^®^ (Medtronic Zomed, Jacksonville, FL, USA) to reduce dead space under the NSF and control bleeding. Postoperative lumbar drainage was performed when large posterior fossa defect was created, or when the onlay fascial graft was not tightly placed within the bone margins.

### 2.4. Data Collections and Outcomes

The demographics assessed and extracted from medical records included age, sex, body mass index (BMI), pathologic diagnosis, tumor size at diagnosis, duration of the surgery, history of radiotherapy or gamma knife radiosurgery, revision cases, postoperative lumbar drainage, and follow-up period. Depending on the origin of the tumor, the skull base defect locations were also checked as suprasellar, posterior fossa, and anterior skull base. The primary endpoints in this analysis were the rate of postoperative CSF leak and meningitis.

### 2.5. Statistical Analysis

All statistical analyses were performed using IBM SPSS statistics version 25, and statistical significance was set at *p* < 0.05. Continuous variables are presented as mean (standard deviation, SD) and categorical variables as numbers (percentages, %). The Kruskal–Wallis test, Chi-Square test, and Fisher’s exact test were conducted. In addition, Mann–Whitney test, Chi-Square test, and Fisher’s exact test were performed to compare according to the presence or absence of postoperative complications as a reference for risk factors that are more likely to cause CSF leak and meningitis. To determine the relevant predictors for postoperative complications, multivariate analysis using logistic regression analysis was performed.

## 3. Results

### 3.1. Patient Demographics

A total of 193 patients who had high-flow CSF leaks during EEA and underwent multilayer reconstruction with an NSF for skull base reconstruction were analyzed in this study. The baseline characteristics, variables related to surgery, and outcomes are shown in Table 1. The mean age at diagnosis was 48.9 years, and 86 (44.6%) patients were male. The mean BMI at diagnosis was 24.4 ± 4.0 kg/m^2^. The most common pathology was craniopharyngioma (*n =* 69, 35.8%), followed by tuberculum sellae meningioma (*n =* 43, 22.3%). Other pathologic diagnoses included 37 pituitary adenomas (19.2%), 11 chordomas (5.7%), 5 olfactory groove meningiomas (2.6%), and 28 types of other tumors (14.5%), including 10 olfactory neuroblastomas, 3 epidermoid cysts, 2 chondrosarcomas, 2 sinonasal squamous cell carcinomas, 2 schwannomas, and 1 each of immature teratoma, mature teratoma, arachnoid cyst, sinonasal adenocarcinoma, medulloblastoma, xanthogranuloma, metastatic adenocarcinoma, gauzoma, and retinoblastoma. The mean tumor diameter at diagnosis was 30.4 ± 10.0 mm, and 24 (12.4%) patients underwent revision surgery. Nine patients (4.7%) had previous radiation therapy and 10 patients (5.2%) had previous gamma knife radiosurgery.

The highest location of the defect opening to the CSF space after tumor removal was in the suprasellar region (162, 83.9%). Thirteen (6.7%) defect openings were in the posterior fossa and 18 (9.3%) in the anterior skull base, such as in olfactory neuroblastoma or meningioma at the olfactory groove. An autologous fat graft was inserted into the intradural dead space in 40 patients (20.7%). Banked fascia lata was used in 102 (52.8%) patients, ADM in 48 (24.9%) and autologous fascia lata in 43 (22.3%) patients. Postoperative lumbar drainages were placed in 111 (57.5%) patients and maintained for 4.8 ± 2.2 days. The mean follow-up period was 3.5 years (from 1 month to 8.0 years).

### 3.2. Surgical Outcomes and Intergroup Differences

**Figure 1:**
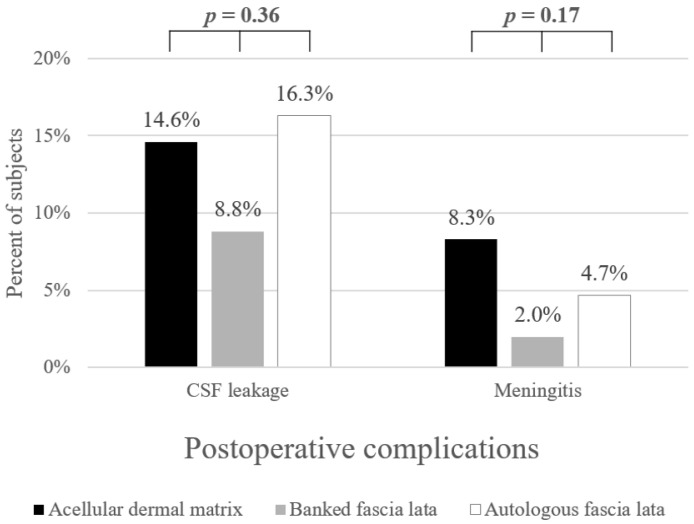
Postoperative complication rate according to graft materials.

Among the 193 patients, postoperative CSF leak occurred in 23 (11.9%) patients, and 8 (4.1%) developed postoperative meningitis (Table 1). Postoperative CSF leak occurred on mean 12.8 ± 7.9 days after surgery, and there was 1 CSF leak that developed after 1 month postoperatively (range 3–37 days). In 91.3% of cases with postoperative CSF leak, endoscopic repair was required, and the remaining 2 patients required treatment with lumbar drainage and absolute bed rest (1 patient each in ADM and autologous fascia lata group). Postoperative meningitis occurred on mean 12.1 ± 4.9 days postoperatively (range 4–17 days). There were no significant differences in the relevant variables, such as age (*p =* 0.95), sex (*p =* 0.33), BMI (*p =* 0.10), tumor size (*p =* 0.42), time taken for surgery (*p =* 0.63), previous radiation (*p* = 0.31) or previous gamma knife (*p* = 1.00) history, revision cases (*p =* 0.15), and diaphragmatic/dural defect location (*p =* 0.52) among the three groups (Table 2). As a surgical outcome, no significant difference in the occurrence of postoperative CSF leak (*p =* 0.36) or meningitis (*p =* 0.17) was observed among the three groups (Figure 1).

### 3.3. Risk Factor Analysis for Postoperative CSF Leak or Meningitis

Variables were compared according to the presence or absence of postoperative complications as a reference for risk factors that are more likely to cause CSF leak and meningitis (Table 3 and Table 4, respectively). There was no significant difference in preoperative parameters, defect location, graft type, and postoperative management according to postoperative complications. There were no significant risk factors for postoperative CSF leak or meningitis, even following multivariate analysis.

## 4. Discussion

Endoscopic skull base surgery is associated with a relatively lower morbidity than craniotomy and easier access to the skull base lesions. However, postoperative CSF leak and meningitis have been the most-criticized complications. The pathway from the sinonasal cavity to the subdural or subarachnoid space must be water-tightly sealed. High-flow CSF leaks commonly occur during resection of craniopharyngiomas, tuberculum sellar/olfactory groove meningiomas, giant pituitary adenomas, clival chordomas, and olfactory neuroblastomas. Many reconstruction methods have been introduced for high-flow CSF leak repair. Previous international consensus statement strongly recommended use of vascularized reconstruction in the presence of large dural defects and high-flow CSF leaks to reduce postoperative CSF leaks [14]. Among vascularized flaps, NSF is the most widely used intranasal flap to provide a sufficient length, width, and rotation range. In addition, multilayer reconstruction techniques using various graft materials have been introduced to reduce the CSF flow rate by the closure of large dural defects and to provide a tight barrier to the paranasal sinus by combining with NSF [5,15,16,17,18]. Autologous fascia lata has been reported as the most used and effective graft material for multilayer reconstruction [15,19,20]. However, more time is required for surgery to harvest fascia lata, and additional wound-related problems may occur. ADM and banked fascia lata with similar properties to dura could be as alternatives. In this topic, three sheet-types of free grafts were performed by inlay and onlay methods for layered dura reconstruction with the NSF in case of high-flow CSF leaks. As there were concerns about the risk of infection or slower healing rate for allografts or ADMs, we directly compared the postoperative outcomes of sheet-type graft materials with similar features [21,22,23]. There was no significant difference in the occurrence of postoperative CSF leak and meningitis among the cases of ADM, banked homologous fascia lata, and autologous fascia lata.

Various techniques using graft materials are available for water-tight skull base reconstruction. “Gasket-seal” method or button-type closure can be performed to provide rigid buttress or to prevent the migration of graft material into intracranial space [24,25]. A direct dural suturing with fascial graft has also been reported for more intuitive defect closure [5]. In this study, double-layered reconstruction was performed in an inlay–onlay fashion using sheet-type grafts, followed by NSF. In order to directly compare the outcomes of three types of materials, we conducted a comparative analysis only on the inlay-onlay method, with as few additional techniques as possible for grafts. Therefore, we believe our results are meaningful as large-series data with consistent surgical techniques that can support the use of non-autologous materials. In addition, 91.3% (21 of 23) patients with postoperative CSF leak required endoscopic repair surgery. During wound exploration, most of the three types of grafts were well-maintained in the inlay–onlay form, and no graft displacement or degeneration was observed.

As reported in one meta-analysis [26], there was no significant difference in the incidence of postoperative CSF leak when comparing autologous and synthetic grafts for endoscopic anterior skull base reconstruction with intraoperative CSF leak, but synthetic grafts were associated with a significantly reduced risk of postoperative meningitis. However, in a previous study, procedures combining vascularized flaps, such as NSFs, were excluded, and banked fascia lata was not included in the non-autologous graft group.

Furthermore, this study only included high-flow CSF leaks following EEA, and the incidences of postoperative CSF leaks and meningitis were 11.9% and 4.1%, respectively; these figures were relatively higher than those reported in previous studies [4,5,12,26]. In our opinion, this is a valuable comparison because high-flow CSF leaks are difficult to deal with following endoscopic skull base reconstruction, and only 57.5% of total patients underwent postoperative CSF diversion.

ADM, being a homologous dermal graft, does not require autologous donor tissue harvesting, so there is no worry about additional wounds or donor-site complications. In addition, since the viable cells are removed, there is little risk of infection, and because the cellular components of the epidermal and dermal layers are removed, the risk of cell-mediated rejection is small [10,27,28]. ADM was reported to be an effective and safe material for skull base reconstruction, and revascularization within the matrix was also observed in histologic review [29,30,31]. The Alloderm^®^ or Megaderm^®^ used in this study was flexible and its thickness was variable, so it was easy to implant into dural defects. However, in our institution, the Alloderm^®^ and Megaderm^®^ are 2.7 and 2.2 times more expensive than banked fascia lata, respectively. Although ADM can be relatively expensive and has slower tissue growth than autologous grafts [21], the outcomes, such as postoperative CSF leak and meningitis, were not inferior to those of autologous fascia grafts.

Banked fascia lata has been widely used in various fields, such as general, orthopedic, gynecologic, or ophthalmologic surgery [22,32,33]. The effectiveness and long-term safety for skull base reconstruction have also been introduced [4,12,34]. To reduce the risk of infection, gamma irradiation should be performed; however, it could cause decreased bioavailability and weaken engraftment with surrounding tissues [12]. Nevertheless, according to this study, there was no significant difference between the postoperative outcomes of autologous fascia lata and banked fascia lata. Therefore, even if intraoperative high-flow CSF leak occurs, banked fascia lata is sufficiently applicable to the dura defect, adaptable to the surrounding tissues, and associated with satisfactory results. However, the availability and price of banked fascia lata may vary depending on the institution.

In this study, we analyzed the clinical outcomes of three different sheet-type graft materials when a high-flow CSF leak was encountered. There have been no studies comparing the outcomes of three types of grafts in cases of high-flow CSF leaks. The complication rate of each group seems different, but since the number of meningitis and postoperative CSF leaks in each group is tiny, there does not seem to be a statistical difference (Figure 1). However, both non-autologous materials had fewer postoperative CSF leaks than autologous fascia lata. Meningitis also occurred less frequently in the banked fascia lata and slightly more frequently in ADM than in autologous ones, so we believe the justification for using non-autologous materials is secure. These alternatives could be safely used, without the risk of additional donor site morbidities, and the difficulty of manipulation during reconstruction was similar to that of autologous fascia lata. In addition, postoperative meningitis occurred within one month of surgery in all three groups, and there was no additional late-onset meningitis during the long term follow-up period up to 8 years. Hence, considering the additional donor-site wound, cost, availability of banked tissue at the hospital, and patients’ preference, it will be possible to select an appropriate fascial graft for the situation.

Several limitations of the study should be reviewed. First, there could be selection bias because this was not a randomized trial. However, graft selection was chiefly determined before the beginning of surgery and there was no significant difference among the groups regarding demographic data that could influence the surgical results. Therefore, the risk of selection bias could be lowered. Second, tumor pathology and defect location heterogeneity may bias the surgical outcome. However, there was no difference in defect location among the three graft groups. Additionally, there was no significant difference in postoperative CSF leaks according to defect location, but 13.0% in suprasellar lesion, 7.7% in posterior fossa lesion, and 5.6% in anterior skull base defect. However, since the number of tumors located in the posterior fossa and anterior skull base is relatively small, 13 and 18, respectively, further large-scale studies for these defect sites will help to evaluate the topics related to this study.

## 5. Conclusions

This large-series study in a single institution suggests that ADM or homologous banked fascia lata could be used instead of autologous fascia lata for multilayer reconstruction of high-flow CSF leaks in the EEA. Furthermore, these alternative non-autologous materials did not increase the postoperative complications and had no additional donor-site morbidities.

## Figures and Tables

**Table 1:** Baseline characteristics.

	All Patients
Number of patients	193
Age, year (range)	48.9 (4–84)
Sex, male	86 (44.6)
BMI, kg/m^2^	24.4 ± 4.0
Pathology	
Craniopharyngioma	69 (35.8)
Meningioma (tuberculum sellae)	43 (22.3)
Pituitary adenoma	37 (19.2)
Chordoma	11 (5.7)
Meningioma (olfactory groove)	5 (2.6)
Etc *	28 (14.5)
Tumor size, mm	30.4 ± 10.0
Time of surgery, min	284.2 ± 94.2
Previous radiation therapy	9 (4.7)
Previous gamma knife radiosurgery	10 (5.2)
Revision surgery	24 (12.4)
Defect location	
Suprasella	162 (83.9)
Posterior fossa	13 (6.7)
Anterior skull base	18 (9.3)
Graft	
Acellular dermal matrix	48 (24.9)
Homologous graft	102 (52.8)
Autologous graft	43 (22.3)
Postoperative lumbar drain	111 (57.5)
Follow-up period, years	3.5 ± 1.9
Postoperative CSF leak	23 (11.9)
Postoperative meningitis	8 (4.1)

Abbreviation. BMI: body mass index, CSF: cerebrospinal fluid. Data are presented as *n* (%) or mean ± SD. * 10 Olfactory neuroblastoma, 3 epidermoid cyst, 2 chondrosarcoma, 2 sinonasal squamous cell carcinoma, 2 schwannoma, immature teratoma, mature teratoma, arachnoid cyst, sinonasal adenocarcinoma, medulloblastoma, xanthogranuloma, metastatic adenocarcinoma, gauzoma, retinoblastoma.

**Table 2:** Intergroup differences in various factors according to graft in dura reconstruction.

	Acellular Dermal Matrix	Banked Fascia Lata	Autologous Fascia Lata	*p*-Value
Number of patients	48	102	43	
Age, year (range) *	48.5 (9–84)	49.5 (6–81)	48.1 (4–77)	0.95
Sex, male ^†^	17 (35.4)	48 (47.1)	21 (48.8)	0.33
BMI, kg/m^2^ *	23.8 ± 3.2	24.3 ± 4.2	25.4 ± 4.4	0.10
Tumor size, mm *	31.0 ± 9.9	29.6 ± 9.6	31.6 ± 10.9	0.42
Time of surgery, min *	268.7 ± 92.3	288.3 ± 92.1	291.7 ± 101.3	0.63
Previous RT ^‡^	1 (2.1)	4 (3.9)	4 (9.3)	0.31
Previous GKS ^‡^	2 (4.2)	6 (5.9)	2 (4.7)	1.00
Revision surgery ^†^	4 (8.3)	11 (10.8)	9 (20.9)	0.15
Defect location ^‡^				0.52
Suprasella	44 (91.7)	82 (80.4)	36 (83.7)	
Posterior fossa	1 (2.1)	9 (8.8)	3 (7.0)	
Anterior skull base	3 (6.3)	11 (10.8)	4 (9.3)	
Postoperative L-drain ^†^	18 (37.5)	66 (64.7)	27 (62.8)	0.005
L-drain duration (days) *	4.9 ± 1.4	4.9 ± 2.4	4.5 ± 2.3	0.57
Postoperative CSF leakage ^†^	7 (14.6)	9 (8.8)	7 (16.3)	0.36
Postoperative meningitis ^‡^	4 (8.3)	2 (2.0)	2 (4.7)	0.17

Abbreviations. BMI: body mass index, RT: radio therapy, GKS: gamma knife radiosurgery, L-drain: lumbar drainage, CSF: cerebrospinal fluid. Data are presented as *n* (%) or mean ± SD. Statistical significance (bold) was defined as *p* < 0.05. *p*-value used by * Kruskal–Wallis test, ^†^ Chi-Square test, and ^‡^ Fisher’s exact test for comparing groups.

**Table 3:** Comparison of various factors according to postoperative CSF leak (Univariate analysis).

	Yes (23)	No (170)	*p*-Value *
Age, year (range)	45.5 (8–77)	49.4 (4–84)	0.50
Sex, male, %	14 (60.9)	72 (42.4)	0.09
BMI, kg/m^2^	24.9 ± 3.7	24.4 ± 4.1	0.39
Pathology, yes, %			
Craniopharyngioma	9 (39.1)	60 (35.3)	0.72
Meningioma (tuberculum sellae)	5 (21.7)	38 (22.4)	0.95
Pituitary adenoma	5 (21.7)	32 (18.8)	0.78
Chordoma	1 (4.3)	10 (5.9)	1.00
Meningioma (olfactory groove)	1 (4.3)	4 (2.4)	0.47
Etc	2 (8.7)	26 (15.3)	0.54
Tumor size, mm	32.2 ± 11.6	30.1 ± 9.7	0.41
Time of surgery, min	251.1 ± 88.3	288.7 ± 94.3	0.10
Previous RT, yes, %	0 (0)	9 (5.3)	0.60
Previous GKS, yes, %	1 (4.3)	9 (5.3)	1.00
Revision surgery, yes, %	5 (21.7)	19 (11.2)	0.17
Defect location, yes, %			0.81
Suprasella	21 (91.3)	141 (82.9)	
Posterior fossa	1 (4.3)	12 (7.1)	
Anterior skull base	1 (4.3)	17 (10.0)	
Graft, yes, %			0.36
Acellular dermal matrix	7 (30.4)	41 (24.1)	
Allograft	9 (39.1)	93 (54.7)	
Autograft	7 (30.4)	36 (21.2)	
Postoperative lumbar drain, yes, %	12 (52.2)	99 (58.2)	0.58

Abbreviations. CSF: cerebrospinal fluid, BMI: body mass index, RT: radio therapy, GKS: gamma knife radiosurgery. Data are presented as *n* (%) or mean ± SD. Statistical significance was defined as *p* < 0.05. * *p*-value using Mann–Whitney test, Chi-Square test, and Fisher’s exact test.

**Table 4:** Comparison of various factors according to postoperative meningitis (univariate analysis).

	Yes (8)	No (185)	*p*-Value *
Age, year (range)	39.4 (8–63)	49.4 (4–84)	0.16
Sex, male, %	4 (50)	82 (44.3)	1.00
BMI, kg/m^2^	23.6 ± 2.8	24.5 ± 4.1	0.59
Pathology, yes, %			
Craniopharyngioma	4 (50)	65 (35.1)	0.46
Meningioma (tuberculum sellae)	2 (25)	41 (22.2)	1.00
Pituitary adenoma	2 (25)	35 (18.9)	0.65
Chordoma	0 (0)	11 (5.9)	1.00
Meningioma (olfactory groove)	0 (0)	5 (2.7)	1.00
Etc	0 (0)	28 (15.1)	0.61
Tumor size, mm	29.3 ± 6.4	30.4 ± 10.1	0.96
Time of surgery, min	237.4 ± 80.4	286.2 ± 94.4	0.21
Previous RT, yes, %	0 (0)	9 (4.9)	1.00
Previous GKS, yes, %	0 (0)	10 (5.4)	1.00
Revision surgery, yes, %	2 (25.0)	22 (11.9)	0.26
Defect location, yes, %			1.00
Suprasella	8 (100)	154 (83.2)	
Posterior fossa	0 (0)	13 (7.0)	
Anterior skull base	0 (0)	18 (9.7)	
Graft, yes, %			0.17
Acellular dermal matrix	4 (50.0)	44 (23.8)	
Allograft	2 (25.0)	100 (54.1)	
Autograft	2 (25.0)	41 (22.2)	
Postoperative lumbar drain, yes, %	4 (50.0)	107 (57.8)	0.73

Abbreviations. BMI: body mass index, RT: radio therapy, GKS: gamma knife radiosurgery. Data are presented as *n* (%) or mean ± SD. Statistical significance was defined as *p* < 0.05. * *p*-value using Mann–Whitney test, Chi-Square test, and Fisher’s exact test.

## Data Availability

Data-sharing not applicable.
